# The role of chromogranin A cleavage products in onset of type 1 and 2 diabetes

**DOI:** 10.3389/fcdhc.2026.1812936

**Published:** 2026-04-13

**Authors:** Gustaf Christoffersson, Elke M. Muntjewerff

**Affiliations:** 1 Department of Medical Cell Biology, Uppsala University, Uppsala, Sweden; 2 Science for Life Laboratory, Uppsala University, Uppsala, Sweden

**Keywords:** catestatin, chromogranin A, diabetes type 1, diabetes type 2, pancreastatin, serpinin, vasostatin, WE14

## Abstract

Chromogranin A (CgA) is a pro-hormone widely expressed in neuroendocrine tissues and elevated in both type 1 (T1D) and type 2 diabetes (T2D). Its diverse biological effects arise from proteolytic cleavage into six bioactive peptides: vasostatin I and II (VS-I/II), chromofungin (CHR), pancreastatin (PST), catestatin (CST), WE-14, and serpinin—each exerting distinct and sometimes opposing functions. This review covers current knowledge on the activity, circulating levels, and mechanistic roles of these peptides in diabetes pathogenesis and progression. Evidence indicates contrasting peptide profiles in T1D and T2D: PST levels are elevated in T2D and promote inflammation, gluconeogenesis, and insulin resistance, whereas CST levels are reduced and exert anti-inflammatory and insulin-sensitizing effects. In T1D, CST and VS-I are increased early after diagnosis, with VS-I and WE-14 functioning as autoantigens that drive autoreactive T cell responses. Knockout mouse models further demonstrate that loss of CgA or CST profoundly alters glucose homeostasis, macrophage polarization, catecholamine release, and diabetes susceptibility. Emerging data highlight a complex nerve–immune–endocrine axis through which CgA peptides regulate metabolic and inflammatory pathways. Collectively, CgA-derived peptides represent promising biomarkers and therapeutic targets, though further translational studies remain essential to define their diagnostic and clinical potential.

## 1 Introduction

High levels of the pro-hormone chromogranin A (CgA) are associated to neuroendocrine tumors and many metabolic, cardiovascular, inflammatory and autoimmune diseases (e.g. inflammatory bowel disease, rheumatoid arthritis) (1). High levels are also found in the saliva and blood of individuals with type 2 diabetes (T2D) and type 1 diabetes (T1D) (2–5). CgA is produced and released by neurons and (neuro)endocrine cells, such as enteroendocrine cells in the gut, chromaffin cells in the adrenal medulla and alpha (α) and beta (β) cells in the pancreas (1). It's striking that elevated CgA levels are detected in various disease types despite varying disease mechanisms. This variability is probably due to the fact that CgA is cleaved into bioactive cleavage products based on the factors in the environment (6). CgA can be cleaved in storage vesicles within the cell or extracellularly upon release by various proteases including furin, prohormone convertases (PC) 1 and 2, cysteine protease cathepsin L (CTSL), the serine proteases plasmin, kallikrein and thrombin (7). Cleavage can result in the six bioactive peptides vasostatin (VS) I or II, chromofungin (CHR), pancreastatin (PST), catestatin (CST), WE-14 and/or serpinin. Since all six bioactive cleavage products have their own specific modes of function, which can even be opposing, this could drive diseases. Although it is clear from literature that CgA plays an important role in regulating different homeostatic processes (1, 7, 8), the knowledge on how its bioactive cleavage products are contributing to diseases like T1D and T2D is currently scattered. In this review, we aim to summarize the current findings of the CgA cleavage product functions and their therapeutic potential in the perspective of diabetes and point out the current knowledge gaps with suggestions on how to move the field forward. Understanding the precise roles and balance of CgA cleavage product levels in diabetes progression would help to understand disease development, advance diagnostics and possible treatments for future patients.

## 2 Main part

### 2.1 Chromogranin A polymorphisms in diabetes onset

Sequencing of the *CHGA* gene locus in several human populations shows common and rare genetic variations in the open reading frame and regulatory regions (such as the proximal promotor and 3´UTR) that influence CgA expression and function (9). Variations that may affect function are specifically observed between Asian and White populations, as well as between Iranian and White males and females (9–11). More clinical studies show that the common genetic variations of the *CHGA* gene are associated with hypertension, hypertensive renal disease and T2D (5, 11–13) possibly by effecting sympathetic activity and endothelin (ET-1) secretion (13, 14). These effects are likely the result of affected generation and function of the bioactive CgA cleavage products. In line with this theory, the PST variant Gly297Ser is an identified risk factor for T2D onset in an Indian population (15, 16). However, more research is necessary to determine the exact role of *CHGA* polymorphisms in T1D and T2D onset and to highlight its potential as biomarker.

### 2.2 Activity and blood levels of CgA derived bioactive peptides in diabetes

CgA and its cleavage products can be detected in plasma, serum or saliva from individuals using ELISA or radioimmunoassay (RIA). So far, studies show quite consistent and stable levels in healthy individuals (0.5–5 nM) (17), but it should be taken into account that medication and diet could affect circulating CgA levels (NCT02620696, NCT00166725, NCT03288402). For CgA, increased levels are detected in saliva and blood of individuals with T2D and T1D (2, 3), where for T1D the CgA blood levels rise early on in T1D onset or when gut related complications arise (4, 18). Since CgA is processed and cleaved differently during inflammation, the blood levels, and perhaps most importantly, the balance between the CgA cleavage products might tell us more about the disease progression (6). Therefore, we summarized the currently known CgA cleavage product blood levels in T1D and T2D onset (**Table 1**).

**Table 1:** Overview of the chromogranin A (CgA) cleavage products and their presence in type 1 diabetes (T1D) and/or type 2 (T2D) diabetes.

CgA cleavage product	T1D	T2D	Functions related to diabetes	REFS
Vasostatin I CgA_1-76_	? (↑)	? (↓)	VS-I parts that act as autoantigen in mice: _m_ChgA_10–19, m_CgA_29-42_, _m_ChgA_34–42_ and _m_CgA_36-44_, _m_ChgA_43–52_ VS-I parts that act as autoantigen in human: _h_ChgA_10–19_ and _h_ChgA_43–52_ Inhibits angiogenesis and vasoconstriction, anti-adrenergic	(23–27, 61, 62, 64)
Vasostatin II CgA_1-113_	? (↓)	↓	Promotes angiogenesis, anti-inflammatory	(20, 21) (19)
Chromofungin CgA_47-66_	? (↓)	? (↓)	Anti-inflammatory, antifungal, cardioprotective	(28–30, 32, 33)
Pancreastatin CgA_250-301_	(↑)	↑	Pro-inflammatory, pro-gluconeogenic, anti-insulin, pro-glucagon	(34–37)
Catestatin CgA_352-372_	↑	↓	Anti-inflammatory, antimicrobial, anti-gluconeogenic, pro-insulin, anti-glucagon, anti-adrenergic, pro-angiogenic, affects leukocyte migration	(4, 38–44)
WE-14 CgA_324-337_	? (↑)	? (↓)	Autoantigen	(23, 49–53)
Serpinin CgA_402-439_	?	?	Cardiac function, cardioprotective, regulates granule biogenesis, anti-apoptotic, inhibits angiogenesis	(22, 55–60)

high levels ↑, low levels ↓ or unknown with speculation ? (↑)(↓), and their function including the references (REFS).

#### 2.2.1 Vasostatin levels are low in T2D, but high in T1D

VS-II is seen as an anti-inflammatory peptide since it inhibits monocyte and macrophage recruitment (19). In line with this, plasma VS-II levels are low in individuals with T2D and are associated with poor coronary collateral vessels in diabetic chronic total occlusions patients (20, 21). For autoimmune diabetes, there is limited human data, but in the streptozotocin-induced mouse diabetes model, which mimics T1D by β-cell destruction, VS-II treatment promoted post-ischemia arteriogenesis and angiogenesis following femoral or coronary artery occlusion (20). If VS-II levels would already have been high, this treatment would likely not have had an effect. At the moment its unknown if VS-II levels in mice reflect the VS-II levels in humans with T1D. In contrast, CgA_1-439_, VS-I (CgA_1-76_) and serpinin (CgA_410-439_) inhibit spontaneous and VEGF- or bFGF-induced angiogenesis from HUVECs grown on Matrigel or in the aortic ring model of angiogenesis (22).

For VS-I, levels are increased in mouse autoimmune diabetes models, non-obese diabetic (NOD) mice, and human pancreas treated with inflammatory cytokines (23, 24). Although it remains to be confirmed in human plasma, we may expect that VS-I levels will be increased in individuals with T1D. This may further link to the disease as parts of VS-I (**Table 1**) act as an autoantigen in T1D, which results in the presence of VS-I-specific CD4^+^ and CD8^+^ T cells (23, 25, 26). These T cells are present in the pancreatic draining lymph nodes and islets in NOD mice, where their amounts increase during disease progression (25). The injection of CgA_36–44_ primed CD8^+^ T cells or CgA_29–42_ naïve splenocytes to mice on a NOD/severe combined immunodefiency (SCID) background results in progression to diabetes (25, 26), which suggests that VS-I drives autoimmune diabetes onset. CgA-specific CD8^+^ T cells and CgA_29–42_ autoantibodies are detectable in blood from NOD mice and individuals with T1D (23, 27). In this perspective, it might be useful to screen VS-I, autoantibodies to VS-I or CgA-specific CD8^+^ T cells in the blood of individuals with high risk of developing T1D.

#### 2.2.2 Chromofungin maintains homeostasis, but its role in diabetes is currently unclear

Little is known about CHR in general and currently there is no simple detection method via plasma or saliva available. However, ulcerative colitis (UC) patients display decreased CHR expression in gut biopsies when compared to healthy individuals (28, 29). Moreover, CHR is cardioprotective (30, 31), antifungal (32) and anti-inflammatory (28, 29, 33). CHR supplementation improved cardiac function by negative inotropism without changing coronary pressure on isolated frog and rat hearts, a mechanism which pharmaceuticals for hypertension and chronic heart failure target. Moreover, administration of CHR after a myocardial infarct improves the infarct size and lactate dehydrogenase levels (30, 31). Based on this, CHR seems to play a role in maintaining cardiovascular homeostasis, which would make it an interesting CgA cleavage product to study in the context of diabetes-related cardiovascular complications.

#### 2.2.3 Pancreastatin and catestatin plasma levels and functions are opposite in diabetes

Individuals with T2D and pregnant women with gestational diabetes display higher PST levels than healthy individuals (34) (35, 36). This rise of PST levels correlates with its proposed functions, since it’s been shown to decrease glucose uptake (34), which is affected during T2D. PST is pro-inflammatory, pro-gluconeogenic, inhibits insulin function and promotes glucagon function (34–37).

In contrast to PST, CST levels are low in T2D (38). This is in line with the notion that PST and CST have opposite effects in the regulation of pancreatic islet homeostasis and inflammation (further discussed in section 2.3). CST is known to be anti-inflammatory, anti-gluconeogenic, to promote insulin function, to inhibit glucagon function, be antimicrobial, anti-adrenergic, induce cytokine production, pro- angiogenic, and is chemotactic, but blocks leukocyte migration in an inflammatory environment (C-X-C motif chemokine ligand 2 (CXCL2) or CC-chemokine ligand 2 (CCL2) (4, 38–44).

Individuals with T1D display increased plasma CST levels, especially in the first 3 years after diagnosis (4, 43). There is only one study that looked into PST levels in T1D, which reported a slight increase in basal PST levels and a significant rise in PST levels upon glucose ingestion, when compared to healthy individuals (37). It would be interesting to study also how the balance between PST and CST levels changes during T1D progression.

#### 2.2.4 WE-14 triggers an autoantigenic T cell response in T1D but its blood levels are unknown

There is currently no data available on WE-14 plasma levels in diabetes, which is surprising since it is possible to detect WE-14 plasma levels with commercial ELISA kits (45, 46). For example, WE-14 levels are reported to be increased in individuals with pheochromocytoma before treatment and normal after surgical removal of the tumor (46). It would be interesting to measure WE-14 levels in individuals with high risk of T1D or at early disease onset since WE-14 triggers an autoreactive CD4^+^ T cell response during disease onset in T1D individuals and NOD mice (47, 48). Although WE14 is mostly mentioned for its properties as an autoantigen in T1D, it seems like VS-I (also an autoantigen) footpad injections to NOD mice induces stronger T cell proliferation in the draining popliteal lymph nodes than WE-14 (23, 49). It can simply be due to the fact that WE-14 was discovered earlier than VS-I, as a result of improved detection methods, or that WE-14 might indeed trigger a stronger autogenic response than VS-I by forming a hybrid-insulin peptide (HIP) or post-translational modifications inducing a stronger response then wildtype WE-14 only (49–53). Regardless of the immunogenicity, measuring WE-14 blood levels or HIP-reactive T cells during T1D onset could serve as a biomarker (54). Based on this knowledge, we would expect WE-14 levels to be high in T1D and low in T2D.

#### 2.2.5 Serpinin affects heart function and granule biogenesis, but its role in diabetes is unknown

At the moment we know very little about serpinin, this might be due to its subsequent processing into various serpinin peptides including full serpinin, pGlu-serpinin and serpinin-RRG. The serpinins seem involved in heart function (myocardial contractility), inhibits angiogenesis, regulate granule biogenesis in (neuro) endocrine cells and have an anti-apoptotic effect on neurons and endocrine cell lines (22, 55–60). To exert its effect on the heart the pGlu-serpinin and full serpinin require the β1-adrenergic receptor (56), which is opposite of CST and VS-I which seem to induce anti-adrenergic cardioinhibitory effects (44, 61, 62). This interplay of CST, VS-I and serpinin on cardiac function should be investigated in the same model to understand the balance of their functions in homeostasis and therapeutical potential for individuals with diabetes. There is currently no data on serpinin plasma levels available, but serpinin has been detected in endocrine cells and organs (heart, nerve fibers in the skin, eye, dental pulp) via immunoreactivity assays, customized ELISAs and a commercially available ELISA kits (56–58, 63). Thus, more research is necessary to understand the function of serpinins and their therapeutical potential.

**Figure 1:**
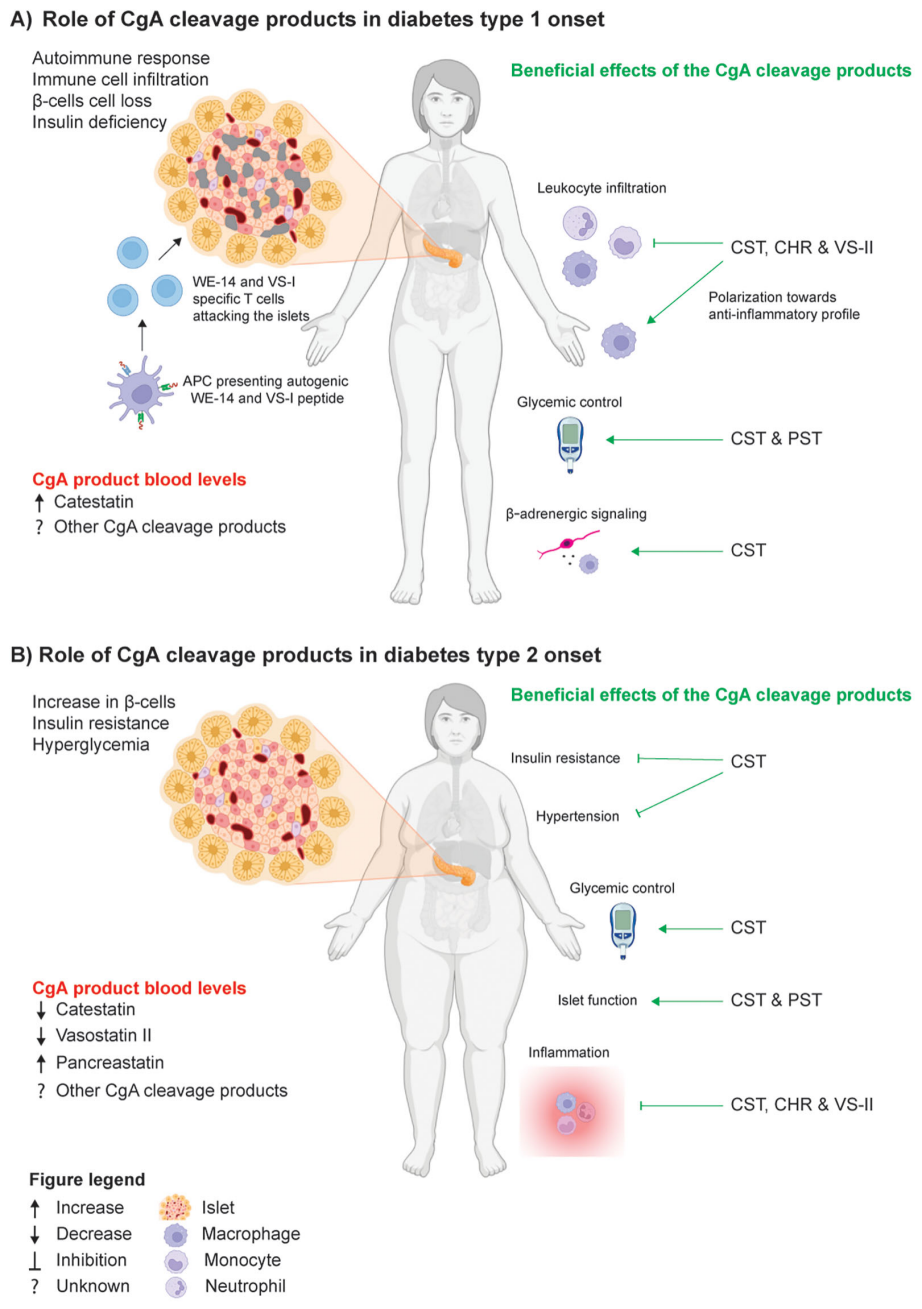
The role of CgA cleavage products in diabetes onset. **(A)** Diabetes type 1 (T1D) is characterized with an autoimmune response which results in islet immune cell infiltration (incl. autogenic WE-14 and vasostatin (VS)-I T cells), β-cell loss and insulin deficiency. Individuals with T1D display high catestatin (CST) blood levels, but the levels of other CgA cleavage products are currently unknown. Beneficial effects of the CgA cleavage products (in green) are observed on 1) leukocyte infiltration and macrophage polarization by CST, chromofungin (CHR) and VS-II 2) Glycemic control by CST and pancreastatin (PST) 3) Interference in nerve-immune communication via β-adrenergic signaling by CST **(B)** Diabetes type 2 (T2D) is characterized with an increase in β-cells, insulin resistance and hyperglycaemia. Individuals with T2D display low CST and VS-II, but high PST blood levels. Beneficial effects of the CgA cleavage products (in green) are observed on 1) insulin resistance and hypertension via CST 2) Glycemic control by CST 3) Endocrine islet function by CST and PST 4) Inflammation via CST, CHR and VS-II (partly created with BioRender).

Altogether, the contrasting CgA cleavage product levels in T1D and T2D onset already show that they likely play a crucial role in disease onset (**Figure 1**). In the future, the profile of CgA cleavage products in plasma could function as a biomarker to predict diabetes onset and/or follow disease progression.

### 2.3 Diabetes onset is affected by the absence of CgA or CST

The absence of CgA in mice results in major phenotypic changes including increased heart rate, hypertension and heart morphology (increased left ventricular mass and cavity dimensions), increased abdominal fat in the first 6 months of age and elevated plasma adipokines, isoprostane, reactive oxygen species (ROS), neuropeptide Y, plasma and adrenal catecholamines, and adrenal ATP content (65–69). Additionally, CgA-KO mice have smaller islets, altered islet endocrine cell composition, and a lower islet density when compared to WT mice (43, 66). CgA-KO mice also display enhanced insulin sensitivity when maintained on a normal chow diet (NCD) (70, 71), which persisted even after four months of high-fat diet (HFD) (72). Moreover, diet-induced obesity (DIO) via HFD on CgA-KO mice resulted in decreased hepatic gluconeogenic and proinflammatory as well as an increase in anti-inflammatory genes in white adipose tissue and peritoneal macrophages when compared to wildtype (WT) DIO mice (72). Strikingly, crossing the CgA-KO mice with the NOD mouse model does no longer result in the development of T1D (73, 74). In contrast to normal NOD mice, the CgA-KO NOD mice show no signs of insulitis in the pancreas, but they do display lymphocytic infiltrate in the salivary glands similar as to NOD mice (73). CgA-reactive T cells still develop in the absence of CgA similarly as in NOD mice, but the difference is that in the CgA-KO mice the T cells remain naïve (73). This might be due to the lack of the autoantigens WE-14 and VS (23–27, 49–53), and the formation of hybrid-insulin peptides. The question arises what the contribution is of the other CgA cleavage products in the development of T1D and T2D.

Although CgA has six cleavage products, only a knockout mouse for CST has been generated to our knowledge (75, 76). Mice lacking CST show low grade organ inflammation, increased plasma catecholamines, chemokines (CXC-1, CCL-2) and inflammatory cytokines (IL-6, TNF-α, IL-1β), increased anti-inflammatory cytokine IL-10, an impaired epithelial barrier, and less gut microbiota diversity (43, 75–77). From the perspective of diabetes, CST-KO mice are very interesting since they are obese, eat more food, display hypertension and are insulin resistant (75, 76) (43). Moreover, pancreatic islet morphology and composition is affected, where like CgA-KO mice, also CST-KO mice have fewer endocrine cells per islet and a decrease in number of β cells (43).

To learn more about the role of CgA cleavage products in diabetes, researchers use CgA-KO, CST-KO and models for T1D (NOD) or T2D (ob/ob, DIO) either with or without CgA cleavage product, CST/PST/VS-I/VS-II/CHR/serpinin, supplementation.

### 2.4 PST and CST oppositely control endocrine function and insulin sensitivity

*In vivo* glucose tolerance tests (GTT) and pyruvate tolerance tests (PTT) in mice lacking CgA or CST shows affected glucose homeostasis, where deletion of CgA results in increased insulin sensitivity, but deletion of only the CST domain results in decreased insulin sensitivity (43). But which CgA cleavage product is responsible for the increased insulin sensitivity?

Healthy individuals show a decrease in forearm *in vivo* glucose uptake following 20 minute PST supplementation via brachial arterial infusion, but not for CST supplementation (34). Moreover, PST supplementation to CgA-KO mice reverses their insulin sensitivity and inflammatory factors, but does not affect the weight gain when compared to WT DIO mice (72). In line with this, supplementation of PSTv1 (PST-NΔ3: CHGA_276–301_), which lacks PST activity, to WT-DIO mice improved insulin sensitivity and glucose tolerance (72). To confirm if the opposite effects on glucogenesis indeed originate from PST and CST presence, CgA-KO mice where supplemented with PST and/or CST for 4 weeks followed by pyruvate tolerance tests (PTT), which indeed showed increased glucose levels upon PST and decreased glucose levels upon CST supplementation (43). Thus, PST and CST have opposing effects in gluconeogenesis, where PST promotes and CST suppresses gluconeogenesis.

The lack of CST also results in lower basal insulin, C-peptide and glucagon levels, which normalize upon CST supplementation (43, 76). In line with this, glucose stimulation of CST-KO mice does no longer result in a rise in insulin plasma levels when compared to WT and CgA-KO mice (43). Also, suppression of glucagon secretion after glucose stimulation was no longer observed in mice lacking CgA or CST when compared to WT mice (43). These findings are in line with the increased insulin sensitivity in CgA-KO and insulin resistance as observed in CST-KO mice (70, 71, 76). Interestingly, islets lacking CST can produce insulin since *in vitro* high glucose stimulation of pancreatic islets isolated from CST-KO mice does result in major insulin release when compared to WT and CgA-KO mice (43). This discrepancy between *in vitro* and *in vivo* is probably due to the presence of catecholamines *in vivo*, which can inhibit insulin secretion via α_2_-adrenergic receptors on beta cells (78, 79). However, this theory should be tested in the future. The role of catecholamines in the onset of diabetes is discussed in more detail in section 2.4.

Since insulin injection in WT mice results in reduced PST plasma levels when compared to basal levels (68), it is likely that also here PST and CST have opposing effects. Indeed PST supplementation to CgA-KO mice inhibits insulin secretion upon glucose stimulation, while CST supplementation results in normal increased insulin plasma levels (43). Moreover, CST supplementation to DIO mice shows decreased plasma insulin levels, lower expression of gluconeogenic genes (*G6pc* and *Pck1)* and improved insulin sensitivity (76). Normal DIO and CST-KO DIO mice display increased plasma insulin levels after 8 hours of fasting, where supplementation of CST brings the plasma insulin levels down for both mice (76), again showing the importance of CST in controlling insulin sensitivity. These findings may be promising for potential exploitation in treatments for T2D since these patients also display low CST plasma levels that correlate with body weight, insulin, glucose and lipid levels (38).

In summary, PST and CST have opposite effects in islet endocrine function and thereby both play a crucial role in maintaining islet homeostasis. In T1D and T2D the function of the islet is disturbed, in the future the supplementation of CST or PST might be an interesting therapeutic (as discussed in section 2.5) to control gluconeogenesis, insulin and glucagon levels, especially in T2D (**Figure 1**).

### 2.5 CgA cleavage products affect macrophages which interfere in diabetes progression

#### 2.5.1 PST has inflammatory, while VS-II, CST, CHR have anti-inflammatory effects via macrophage polarization and migration

DIO mice are characterized with an inflammatory macrophage profile, but upon CgA depletion the CgA-KO DIO mice display a more anti-inflammatory profile. PST seems to enhance this inflammation since PST supplementation to CgA-KO DIO mice shows an inflammatory profile similar to normal DIO mice (72). Moreover, PST treatment of WT or CgA-KO peritoneal macrophages shows an increase in inflammatory marker expression (*Nos2, Tnfα, Mcp1, Il6*) (72). In contrast, upon CST supplementation, normal DIO mice show reduced monocyte-derived macrophage (F4/80^−^Ly6C^+^) infiltration in the liver (76). CST and PST are known to be chemotactic for leukocytes (42, 72). However, *in vivo* and *in vitro* migration assays show that if CST is present in an inflammatory environment, it blocks migration towards inflammatory chemokines (CCL2, CXCL2) (42), which would explain the observed reduced liver infiltration in DIO mice. Additionally, liver macrophages isolated from DIO mice supplemented with CST *in vivo* or *in vitro* showed a reduction of pro-inflammatory genes (*Emr1, Itgam, Tnfa, Ifng, Nos2, Ccl2*) and an increased expression of anti-inflammatory genes (*Il-10, Clec10a*) when compared to liver macrophages from normal DIO mice (76). Not only CST, but also VS-II and CHR have shown beneficial effects on leukocyte migration and polarization in inflammatory diseases.

Administration of VS-II to atherosclerotic (apolipoprotein E-deficient (ApoE^-/-^)) mice on high-fat diet showed reduced expression of inflammatory and chemoattractant markers (TNF-α, MCP-1, VCAM-1) in aortic tissues (19). Additionally, *in vivo* and *in vitro* migration assays confirmed that VS-II treatment suppresses leukocyte adhesion to the vessel wall and monocyte and macrophage migration (19). For CHR, its intra-rectal (*i.r.*) administration to dextran sulfate sodium (DSS) induced colitis mice has a beneficial effect on colitis progression, which also seems related to the effect on macrophages, monocytes and neutrophils. CHR administration to DSS mice or LPS-treated macrophages *in vitro* resulted in reduced (myeloperoxidase) MPO, C-reactive protein (CRP), monocyte chemoattractant protein-1 (MCP1) protein, NF-κBp65 protein levels, reduced mRNA expression of inflammatory markers (*Tnfα, Il1b, Il6, Tlr4 and Rel*) and macrophage inflammatory factor (*mip1a* and *mip1b*) when compared to PBS or scrambled CHR peptide (sCHR) treatment (29). *In vivo* or *in vitro* CHR-treated peritoneal macrophages of DSS-colitis WT mice show a more anti-inflammatory profile since they express higher levels of *Il10*, arginase-1 (*Arg1*), chitinase-like protein (*Ym1*) and found in inflammatory zone protein (*Fizz1*) (28). Although not tested in a diabetes model, the beneficial effects of CHR and CST on macrophage polarization and migration seem similar. The question arises if they would compete or collide with each other when supplemented at the same time. CHR and CST can both induce a concentration-dependent transient increase of intracellular calcium *in vitro* in human polymorphonuclear neutrophils (PMNs) (33). Addition of both peptides at the same time showed a higher calcium increase than separate peptide stimulation. This suggests that CHR and CST enhance each other’s functions rather than opposing each other (33). More research is necessary to define the exact effect of CHR on the function of neutrophils, monocytes and macrophages. Additionally, it would be very interesting to investigate if this increase in positive effect on inflammation is also observed upon VS-II, CHR and CST treatments in diabetes models.

#### 2.5.2 CST exerts its beneficial effects in diabetes progression via macrophages

Macrophages are known to play a key role in the development of T1D since clodronate treatment of autoimmune diabetic mice (RIP-LCMV-GP and NOD) resulted in reduced T1D onset (80). Strikingly, depletion of macrophages via clodronate treatment in DIO mice supplemented with CST no longer results in improved glucose tolerance and insulin sensitivity (76). Thus, it seems like the macrophages (or other phagocytes that are affected by clodronate treatment) are key players in exerting the beneficial effect of CST in diabetes onset. Some researchers claim that CST can even be produced by macrophages (75), while others show the opposite (6). Nevertheless, it would be possible for the macrophages to receive vesicles containing either CgA or its cleavage products from neurons or (neuro)endocrine cells that can be released in the liver or pancreas to interfere locally (81). Although not tested for diabetes, it is plausible that CST exerts its beneficial effect in diabetes progression via macrophages.

#### 2.5.3 CST interferes in nerve-immune-endocrine communication via catecholamines

Increasing amounts of data from both human histopathological and mouse studies point to an active involvement of neuronal input to the immune environment in T1D onset. Human T1D pancreata show signs of sympathetic neuron loss at early disease onset (82), and even in pre-symptomatic autoantibody-positive individuals (83). In mouse models, interfering with pancreatic sympathetic innervation (80) or neuronal activity to pancreatic draining lymph nodes limits disease onset (84). Some of these effects have been hypothesized to be linked to neurotransmitter input to macrophages (80). The densities of sympathetic nerves and macrophages are not affected in the CST-KO mice. However, nerve-macrophage interactions seem to be less frequent upon CgA or CST deletion (43). CST is known to inhibit catecholamine release as shown on rat PC12 pheochromocytoma cells (cell line from an adrenal gland tumor), primary cultures of adrenal chromaffin cells and noradrenergic neurites (85). In line with this, CST-KO mice display increased plasma levels of catecholamines when compared to WT mice (43). Since pancreatic islets produce CST, this might act as a local regulator of catecholamine release from islet neurons.

At T1D onset, the release of catecholamines from the adrenal gland into the plasma is disturbed which negatively affects glycemic control (86). Since CST levels are high at early T1D disease, CST might play a role here by inhibiting release or affecting the breakdown of catecholamines.

Altogether it seems that CST may exert some of its effects on diabetes onset via a complex nerve-macrophage-endocrine interplay where the control of catecholamine levels plays a key role. Low CST levels result in high catecholamine levels contributing to insulin resistance and hypertension as observed in T2D (38, 75, 76, 87). In contrast, increased CST levels, lower catecholamine levels and elevated nerve growth factor (NGF) levels are observed in T1D (4, 43, 88), which could contribute to increased insulin sensitivity and loss of homeostasis by disruption of nerve-immune communication. However, more research would be necessary to be able to define the effects of CST on catecholamine levels and its contribution to nerve-immune communication in T1D onset. Additionally, the interplay of other CgA cleavage products that can affect macrophages such as PST and CHR should be investigated.

### 2.6 Bioactive CgA derived peptide receptors to execute their physiological function

Although our understanding of the functions of CgA-derived peptides increases, the studies and knowledge on their receptors are still limited.

The peptides CHR and CST can penetrate the bacterial cell wall directly to execute their antibacterial and antifungal function (32, 39, 89). VS-I/II working mechanism seems to include phosphoinositide 3-kinase-dependent eNOS phosphorylation by binding to the cell membrane via phosphatidylserine or other membrane-relevant phospholipids, heparan sulfate proteoglycans and/or neuropilin-1 (90–92). For serpinin there are hints that it executes its effect via a GPCR since it triggers cAMP formation and/or a β1-adrenergic receptor (56, 59). CST has a broad variety of functions and seems to execute its function via muscarinic and nicotinic acetyl choline receptors, G-protein coupled receptors and potentially β-adrenergic receptors (42, 85, 93–97). Since PST has opposite effects of CST it might bind similar receptors, but so far it seems that PST binds glucose regulated protein 78 (GRP78) as well as G-protein coupled receptors, Gα_q/11_ and Gα_i1,2_ (98–100).

In summary, the CgA derived peptides seem to use two mechanisms of action to execute their physiological function. They either penetrate or bind directly to the cell membrane as observed for VS-I/II, CHR and CST (32, 39, 89–92) and/or they bind receptors on the cell surface as seen for serpinin, CST and PST (42, 56, 59, 85, 93–100). Since the physiological function of the peptides have such a wide range of effects, the bioactive peptides might use several different receptors in both potentiating and antagonizing fashions to execute their functions.

### 2.7 Therapeutic potential of CgA cleavage products in diabetes treatment

#### 2.7.1 CgA cleavage product supplementation as medication

Although CST is not approved to use as a treatment in the clinic yet, the findings presented above positions it as a potential treatment avenue for patients with diabetes (**Figure 1**). We know that the administration of CST or PST is not harmful for healthy or diabetic individuals (34, 37). It is also known that hypertension and even changes in islet morphology are reversible upon normalizing blood glucose levels as shown by anti-diabetic drug (insulin or glibenclamide) treatment of bV59M mice, with β-cell dysfunction exposed to long-term hyperglycaemia (101).

Normal CST is not stable for peroral administration, however the analogue retro-inverso (R-I) CST or the CST analogue TKO-10–18 have peroral bioavailability and activity as tested for hypertension and diabetes (102, 103).

#### 2.7.2 Inhibitors of CgA cleavage products as medication

Instead of CST supplementation, it could be beneficial to block CST or PST levels/function as a treatment for individuals with diabetes. A promising drug to block PST function is PSTi8, which seems most effective when administered in low doses for a longer period of time. PSTi8 competes with PST for binding on the GRP78 receptor resulting in enhanced insulin sensitivity and improved glucose homeostasis in mouse models for obesity and T2D (HFD, HFrD and db/db) (104, 105). The autoantigenicity of WE-14 and serpinin could be used to develop better antigen-specific immunotherapy for individuals with T1D or at high risk of developing T1D (106, 107). Altogether, CgA peptides seem promising for the development of new medication for individuals with diabetes. However, more research is necessary to determine the therapeutic potential of CgA cleavage products and inhibitors in humans.

## 3 Discussion

Although the field mostly used mouse models, it is increasingly clear that CgA and its cleavage products are crucial for controlling body homeostasis (**Figure 1**). The lack of certain CgA cleavage products seems to drive important parts of the development of T1D and T2D. Although the field made important discoveries in the past 10 years, the following questions remain to be answered in the future 1) How can we translate the finding from mouse to human? 2) Is it only the lack of the autoantigens (WE-14 and VS-1) that protect CgA-KO NOD mice from the development of autoimmune diabetes? 3) How do the reported inflammatory effects of PST and anti-inflammatory effects of CHR as well as CST contribute to the onset of T1D and T2D? 4) How do the organ and blood levels of all known CgA cleavage products elevate during T1D and T2D development and how are they regulated? What are the pathways and receptors involved in executing their beneficial effects? To answer these questions. future research should include human research using for example plasma samples, human derived islets and clinical studies. Understanding the exact role and balance of CgA cleavage product levels in diabetes progression would help to develop diagnostic assays to follow disease progression and possible treatments by blocking or supplementing CgA cleavage products to treat diabetes in the future.
